# Comparative Cranial Geometric Morphometrics among Wistar Albino, Sprague Dawley, and WAG/Rij Rat Strains

**DOI:** 10.3390/ani14091274

**Published:** 2024-04-24

**Authors:** Duygu Küçük Ağaç, Burcu Onuk, Ozan Gündemir, Murat Kabak, Nicoleta Manuta, Buket Çakar, Maciej Janeczek, Denise Amber Crampton, Tomasz Szara

**Affiliations:** 1 Department of Veterinary, Şiran Mustafa Beyaz Vocational School, Gümüşhane University, 29700 Gümüşhane, Türkiye; duygu.kucuk@gumushane.edu.tr; 2 Department of Anatomy, Faculty of Veterinary Medicine, Ondokuz Mayıs University, 55270 Samsun, Türkiye; burcuonuk@omu.edu.tr (B.O.); mkabak@omu.edu.tr (M.K.); 3 Department of Anatomy, Faculty of Veterinary Medicine, Istanbul University-Cerrahpasa, 34500 Istanbul, Türkiye; 4 Institute of Graduate Studies, Istanbul University-Cerrahpaşa, 34500 Istanbul, Türkiye; nicoleta.manuta@mail.ru (N.M.); buketcakar4@gmail.com (B.Ç.); 5 Department of Biostructure and Animal Physiology, Faculty of Veterinary Medicine, Wrocław University of Environmental and Life Sciences, 50-375 Wrocław, Poland; maciej.janeczek@upwr.edu.pl; 6 Research Centre in Evolutionary Anthropology and Palaeoecology, School of Biological and Environmental Sciences, Liverpool John Moores University, Liverpool L3 3AF, UK; d.crampton@2023.ljmu.ac.uk; 7 Department of Morphological Sciences, Institute of Veterinary Medicine, Warsaw University of Life Sciences-SGGW, 02-776 Warsaw, Poland

**Keywords:** skull, molar teeth, shape variation, veterinary anatomy, taxonomy

## Abstract

**Simple Summary:**

Geometric morphometrics allows the examination of the shape variation of structures. In this study, the skull, mandible, and teeth of three different strains of rats were examined. The results showed that the strains significantly differed in shape. The most important shape difference in the skull was the variation of the cranium from an oval to an elongated structure. In this respect, Sprague Dawley rats showed a more elongated skull, while WAG/Rij rats had a more oval skull. Wistar Albino rats showed a more moderate shape variation. WAG/Rij rats showed very different shapes of the mandible and teeth compared to the other strains. But statistically, it was seen that all strains were completely separated from each other.

**Abstract:**

This research utilizes geometric morphometrics to investigate shape variation in the skull, mandible, and teeth among three rat strains: Wistar Albino (WA), Sprague Dawley (SD), and WAG/Rij (WR). Through the analysis of 48 rats using 2D geometric morphometric techniques, significant differences in their skull morphology were identified. This study indicates a shift from a rectangular to an oval cranial shape across strains, with notable size and morphological variances. Particularly, the WR strain’s skull shape significantly differs from the SD and WA strains, suggesting distinct ecological or genetic pathways. Compared to the skull, mandible shape differences are less pronounced, but still significant. The WR strain exhibits a distinct mandible shape, potentially reflecting ecological adaptations like dietary habits. The teeth shape of WR rats is the most distinct. SD rats consistently exhibited larger sizes in both skull and mandible measurements, while WR rats were notably smaller. Interestingly, sexual dimorphism was not statistically significant in skull and teeth sizes, aligning with findings from previous studies. However, the mandible showed clear size differences between sexes, underscoring its potential for adaptive or behavioral studies. In summary, this study provides a comprehensive analysis of morphological variations in rat strains, highlighting the intricate interplay of size, shape, and ecological factors. These findings lay a foundation for deeper explorations into the adaptive, ecological, or genetic narratives influencing rat morphology.

## 1. Introduction

Rodents are the most diverse mammalian order and are the most frequently used animal group in biomedical research. [1]. Part of the reason is that they have a large number of mutations [2]. Due to these mutations, differences form in their physiological and anatomical structures. Rats are rodents with varying sizes and tail lengths. Most inbred lines used in research today originate from the Wistar Albino line [2]. Rats are used in research in the fields of basic medicine, pharmacology, food, and behavior. There are approximately 400 inbred and 50 outbred lines that have been genetically defined [2]. The Sprague Dawley rat is the most used strain for pharmaceutical research in the United States and Japan [3]. Compared to the laboratory mouse, there are fewer rat strains commonly available and used in biomedical research. Another frequently used rat strain is the well-bred Wistar strain [3]. Initially developed as a model for epilepsy, the WAG/Rij rat was later used in the study of many similar diseases [4]. WAG/Rij rats (Wistar Albino Glaxo from Rijswijk) are an inbred strain of rats with genetic absence epilepsy, a non-convulsive type of epilepsy [5].

The skull consists of many bones, mostly paired, but also unpaired. Cranial bones, also called the neurocranium, are the bones surrounding the cranial cavity. The second group, facial bones or the splanchocranium, surrounds the nasal cavities, paranasal sinuses, and the oral cavity [6,7,8,9].

Shape analysis plays an important role in many biological studies [10,11]. Various biological processes, mutations, and genetic developments cause differences in the morphological shape among individuals [12]. Shape analysis is an approach to explain this morphological diversity [13,14]. Shape analysis can be done with geometric morphometrics, a method based on statistical tools to explain different shapes [11,14]. Geometric morphometrics is a technique to study scale and shape relationships of structures using cartesian geometric coordinates rather than linear, areal, or volumetric variables. Geometric morphometrics is the most current and effective method for examining the morphology of structures, providing insights into their shape, both visually and in terms of enhanced structural definition.

The skull is anatomically complex and phylogenetically diverse. Therefore, it is an important material for examining the role of developmental processes in evolutionary change [12,15]. Variations in skull morphology within the same species have been noted in numerous studies. [16]. Kryštufek et al. [17] conducted shape analysis on rat skulls and revealed skull variations in different rat strains. In another study, Samuel et al. [18] revealed skull variations in two African rodents using geometric morphometry. While these studies contribute to a better understanding of skull morphology in rats, they also play a part in the identification of strains from a taxonomic perspective. Yanoi [19] showed that the brains of both female and male Wistar Albino rats are larger than that of Long-Evans rats and these differences are not related to body weight. Small differences in the overall size of the cranial cavity, and therefore, the brain, may represent larger differences in their components, which may be important in studies of the central nervous system using animal models [20]. Based on these ideas, this study aimed to examine the shape variations of the skull, teeth, and mandible in Wistar Albino, Sprague Dawley, and Wag/Rij rat strains. Shape differences were revealed and distinctive features between strains were examined.

## 2. Materials and Methods

### 2.1. Samples

In this study, a total of 48 rat skulls and mandibles belonging to 3 different strains, Wistar Albino (WA), Sprague Dawley (SD), and WAG/Rij, stored in Ondokuz Mayıs University Faculty of Veterinary Medicine Department of Anatomy were used. All animals were 8 weeks old. Sixteen rats, eight males and eight females, from each strain were examined.

High-resolution images of the skulls were captured using a Canon 500D camera (Canon, Tokyo, Japan).

### 2.2. Data Collection

**Figure 1:**
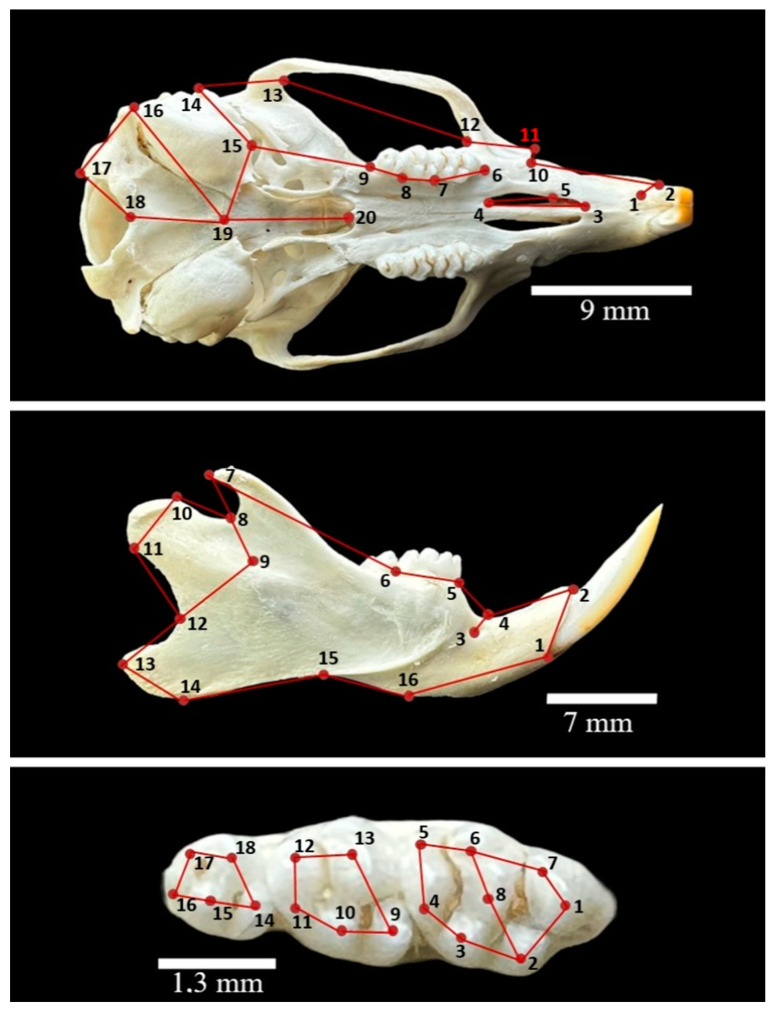
The position of landmarks used to characterize the shape of the ventral skull, labial side of the mandible, and occlusal surface of the upper molars (the labial side is at the top).

We employed a standardized imaging approach for the skull, mandible, and teeth, capturing photos from distances of 5 cm (skull and mandible) and 2 cm (teeth). Consistency was maintained by using the same angle for each sample, with images saved in JPEG format. For landmark analysis, separate “tps” files for each anatomical part (skull, mandible, and teeth) were prepared using tpsUtil (version 1.74) [21] with the TpsDig software (version 2.3) [22]. Twenty landmarks were placed on the ventral view of the skull, 16 on the lateral side of the mandible, and 18 on the occlusal surface of the teeth (Figure 1). Our landmarking methodology was adapted from Kryštufek [17]. A detailed description of the location of landmarks is given in Appendix A.

A wireframe was created by connecting the points. This wireframe was used to show the shape variation that changed as a result of the principal component analysis.

### 2.3. Geometric Morphometrics

Geometric morphometric analysis was performed using ‘M‘orphoJ’’ [23]. Before the analysis, the imported landmark data underwent a Generalized Procrustes transformation [24]. Principal component analysis (PCA) was employed to reduce the number of variables and easier visualization of within-sample variations with the distributions for the first 2 principal components, accounting for the highest proportion of variation, being visualized in graphical format. Mean shapes between groups were obtained. Centroid size values were calculated for 3 groups and the difference between the groups was examined statistically with ANOVA. Centroid size, a common measure in geometric morphometrics, captures an object’s size variation independently of its shape. It is derived as the square root of the sum of the squares of the Euclidean distances from each landmark to the configuration’s centroid. MorphoJ automatically computes centroid size values for each sample. A calibration factor based on pixels was used to obtain the coordinates. Canonical variate analysis (CVA) was used to evaluate how many axes contribute to discriminating between strain groups. Procrustes distances and Mahalanobis distances were calculated to express differences between species in units of standard deviation.

## 3. Results

### 3.1. Size

**Figure 2:**
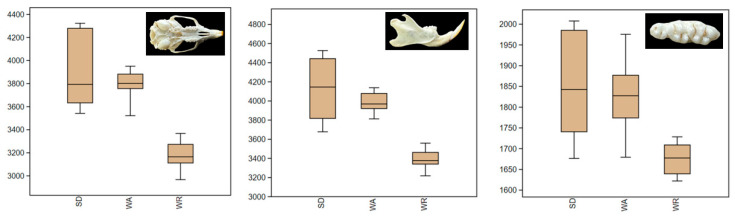
Boxplot with variation in centroid size for the ventral view of the skull, labial side of the mandible, and occlusal surface of the upper molars for three strains (Wistar Albino, WA; Sprague Dawley, SD; WAG/Rij rats, WR). The darker horizontal line is the median, the margins of the boxes represent the first and third quartiles (25th and 75th percentiles), and the horizontal lines in the boxes represent the median (50th percentile).

SD exhibited a notably larger average size variation compared to other strains, with WR being the smallest (Figure 2). The size patterns for the head and mandible were congruent. Interestingly, the tooth measurements for WA exhibited higher variability than the other parameters studied. The ANOVA results showed that the difference between the sample averages of some groups is big enough to be statistically significant (Table 1 and Table 2). WR centroid size values for the skull, mandible, and molar tooth were statistically different from the other two strains (*p* < 0.01). However, the difference between the SD and WA centroid sizes was statistically nonsignificant.

### 3.2. Shape Variation

**Figure 3:**
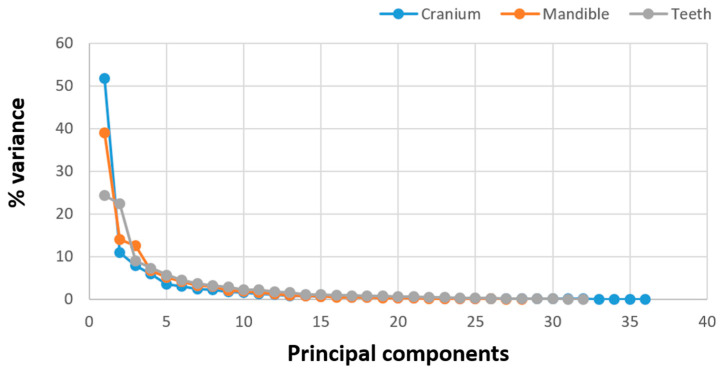
Scree plot of variance (%) accounted for the principal components for the ventral skull, mandible, and teeth.

As a result of the Principal Component Analysis, the number of dimensions was reduced and the most significant Principal Components were extracted. The scree plot showing the component number with eigenvalues for the ventral skull, mandible, and teeth is presented in Figure 3.

**Figure 4:**
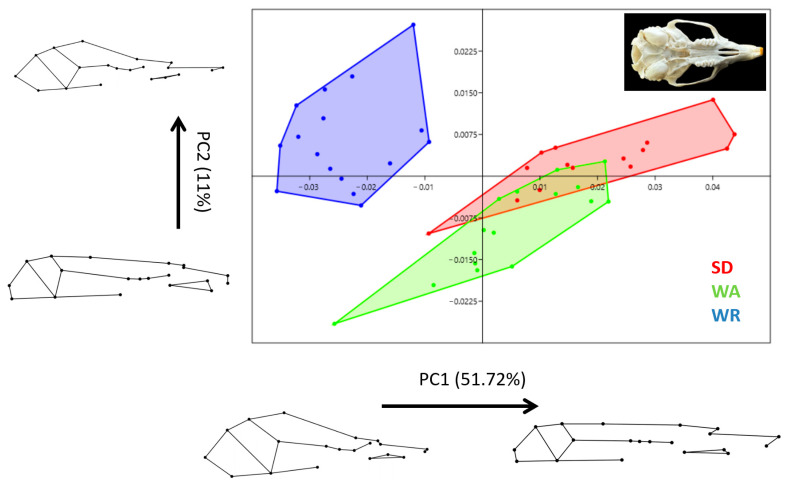
Principal component analysis scatter plot comparing skull morphology of three strains (Wistar Albino, WA; Sprague Dawley, SD; WAG/Rij rats, WR). Wireframe plots describing cranial shape between the negative (at 0.1 scaling factor for PC) and positive (at 0.1 scaling factor for PC) values of PC1 and PC2.

The Procrustes superimposition of the 20 skull landmarks resulted in 36 shape variables. PC1 explained 51.72% of the total variation, and PC2 explained 11.0%, cumulatively explaining 62.72% of the morphological variance (Figure 4). A higher PC1 value indicated a more elongated skull, whereas a lower PC1 value represented a more oval-shaped skull with a wider cranial base and more prominent zygomatic arches. An increasing PC2 value represented a narrower facial part of the skull. Based on the PC1 and PC2 results, the WR rats showed distinct shape differences compared to the other two strains with a wider base, particularly in its cranial part, and a narrower bony palate. The SD and WA rats had similar PC1 values.

**Figure 5:**
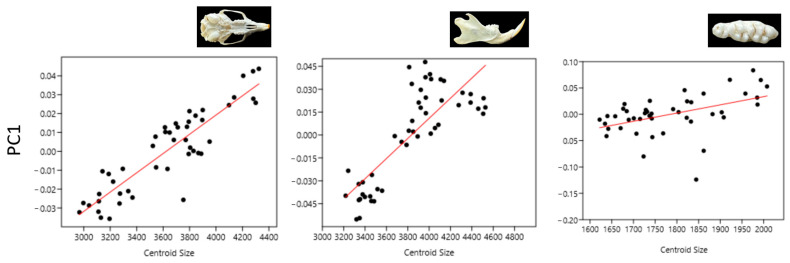
Effect of PC1 values on centroid size.

Size explained 79.9% of the shape variation by PC1, and this was significant (*p* < 0.0001) (Figure 5). As such, a pronounced allometric factor was observed when comparing rat skull morphologies for PC1.

**Figure 6:**
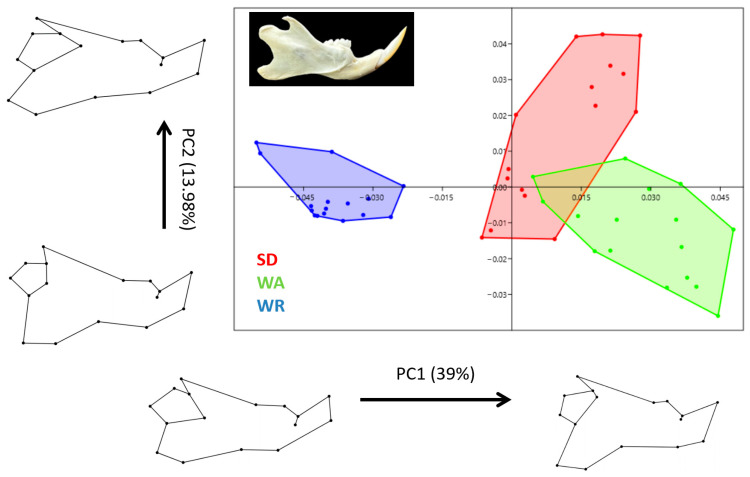
Principal component analysis scatter plot comparing mandible morphology of three strains (Wistar Albino, WA; Sprague Dawley, SD; WAG/Rij rats, WR). Wireframe plots describing cranial shape between the negative (at 0.1 scaling factor for PC) and positive (at 0.1 scaling factor for PC) values of PC1 and PC2.

For the mandible, PC1 explained 39.0% of the total variation, and PC2 explained 14.0%, together explaining 52.98% of the morphological variability (Figure 6). An increased PC1 value represented a more oval and taller mandible, while a negative PC1 value represented an elongated mandible. At a positive PC1 value, the ventral edge of the mandible was more curved and the angular process was more ventral. In increasing PC2, the angular process was more caudal. For PC1, the shape of WR rats significantly differed from those of the other strains. PC2 values of SD rats were higher than other strains. Except for two samples of WA rats, the others had different shapes compared to SD rats. In addition, 64.9% of the shape described by PC1 is predicted by size, which is also significant (*p* < 0.0001).

**Figure 7:**
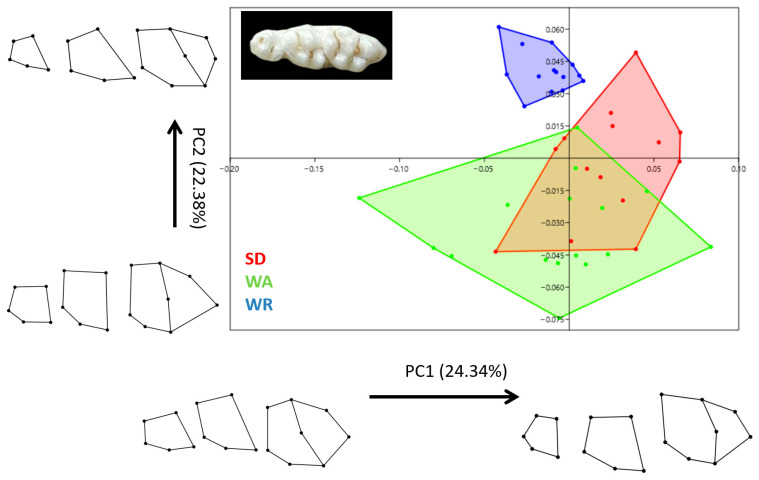
Principal component analysis scatter plot comparing teeth morphology of three strains (Wistar Albino, WA; Sprague Dawley, SD; WAG/Rij rats, WR). Wireframe plots describing cranial shape between the negative (at 0.1 scaling factor for PC) and positive (at 0.1 scaling factor for PC) values of PC1 and PC2.

For the teeth, the first two principal components explain 46.7% of the shape variation. With increased PC1 values, the anterior part of the tooth became narrower (Figure 7). With increasing PC2 values, the anterior part of the tooth became blunter. For negative PC2 values, the anterior part of the tooth was more pointed. PC2 differentiated WR from WA rats, but it overlaps with SD rats in the teeth shape. SD and WA rats, on the other hand, showed more similar shape variations (see Figure 6). The shape was significantly influenced by size (*p*: 0.0015), with size explaining 21.3% of the variation along PC1. Although the teeth morphology of rats for PC1 was not as strong as found in the skull and mandible.

### 3.3. Canonical Variates Analysis

**Figure 8:**
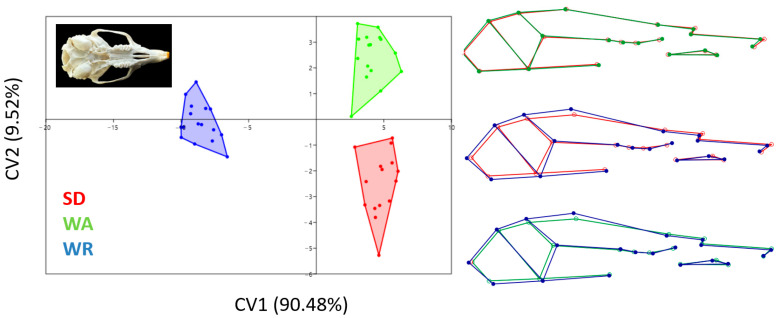
Canonical variates analysis scatter plot comparing skull morphology of three strains (Wistar Albino, WA; Sprague Dawley, SD; WAG/Rij rats, WR). Wireframe plots represent cranial mean shapes for three strains.

According to CV1, WR rats had a different skull shape to other strains (Figure 8). The neurocranium in WR rats is wider than in SD and WA rats. In general, WR rats had a larger skull than the other strains. SD and WA skull shapes were very close to each other. However, according to CV2 (9.52), SD and WA skull shapes were completely separated from each other.

**Figure 9:**
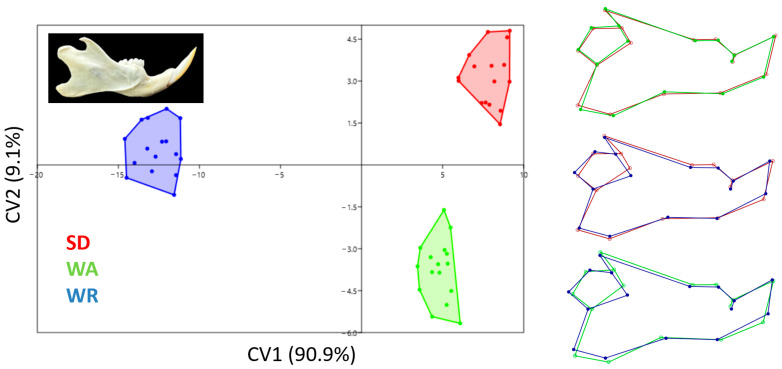
Canonical variates analysis scatter plot comparing mandible morphology of three strains (Wistar Albino, WA; Sprague Dawley, SD; WAG/Rij rats, WR). Wireframe plots represent mandible mean shapes for three strains.

The mandible results were very similar to the skull results (Figure 9). CV1 distinguished the WR mandible from the other strains. The mandibular notch was deeper in WR rats than in other strains. Moreover, the rostral part of the mandibular body in WR was narrower than that of other strains. The mandibular angle was lower in WA rats.

**Figure 10:**
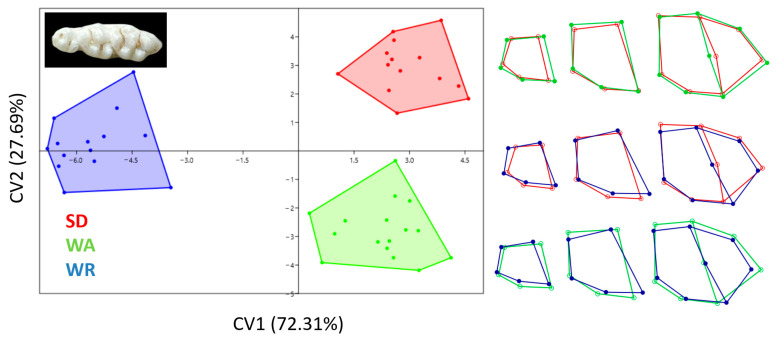
Canonical variates analysis scatter plot comparing teeth morphology of three strains (Wistar Albino, WA; Sprague Dawley, SD; WAG/Rij rats, WR). Wireframe plots represent teeth mean shapes for three strains.

As in previous results, CV1 distinguished WR teeth from other strains (Figure 10). It was seen that all strains differed from each other in terms of the skull, mandible, and teeth.

The Procrustes results between the groups are given in Table 3. The shape of the skull, mandible, and teeth was found to be different between the strains. In terms of the Procrustes distance, the WR group was further away from the other strains. As for the *p* value, all distances between the groups were statistically significant. Additionally, according to the CVA results, the proportion of each group that is correctly classified and the proportions of each group that are incorrectly classified are given in Table 4.

The classification report presented in Table 3 indicates that all classifications for the cranium, mandible, and teeth were correct. However, there were higher misclassification rates between SD and WA rats in the cranium compared to the other pairings. For the mandible, there was a 25% misclassification rate across the entire sample. In teeth, the misclassification rate between SD and WA rats was higher compared to other pairings, similar to the cranium.

## 4. Discussion

Our study highlights clear size differences among rat strains, similar to the results in Kryštufek’s study [17]. Specifically, SD rats were typically larger, while WR rats were the smallest. This consistent size difference, observed in both skull and mandible measurements, suggests possible evolutionary or genetic reasons behind these variations.

The variability exhibited by WA rats in tooth measurements is intriguing. This heightened variability, which contrasts with other strains and parameters, suggests that teeth might be undergoing different evolutionary pressures compared to other skeletal features. Whether this is a result of diet, environmental pressures, or some other ecological factor remains an area ripe for further investigation.

Caumul and Polly [25], examining the morphology of Eurasian marmots, showed that ventral skull shape variation depends primarily on genetic factors, while the shape of the lower jaw is more susceptible to environmental influence. Molars hold an intermediate position.

The ventral skull shape among different rat strains elucidated through our study exhibits a notable differentiation, significantly informed by both size and morphological variances. With PC1 alone accounting for over half of the variance, it is clear that the transition from a more rectangular to an oval skull shape is a pivotal morphological distinction. The fact that with increased PC1 values we approach the occipitalis level of the foramen magnum hints at potential structural or functional implications that warrant further exploration.

In this study, the most shape variation seen in the skull was from a more rectangular to an oval skull shape. In another study conducted on different rat strains, the shape variation of PC1 was similar to this study. In Kryštufek’s et al.’s study, the PC1 shape change revealed a transitional shape variation from an oval skull to a more rectangular skull [17]. The results revealed a clear distinction between the Bandicota and Nesokia strains in the shape of the ventral skull. However, more studies need to be done on this subject to be able to say that skull shape variation in rats is generally like this.

The differentiation of WR from SD and WA rats in the morphospace, guided by the PC1 values, underscores potential ecological or genetic distinctions among these strains. This is quite intriguing considering the genetic similarity between WR and WA rats. Moreover, the overwhelming influence of size, explaining almost 80% of the variance in skull shape by PC1, is a testament to the profound allometric patterns in rat cranial evolution. The WR strains’ distinct skull shape, compared to the similar morphologies of the SD and WA strains, could be indicative of different ecological niches or evolutionary paths. The strong allometric component in shape variations underscores a compelling interplay between size and shape, potentially hinting at specific adaptive, functional, or ecological dynamics influencing these variances.

Differences in the mandibular shape, although significant, are less pronounced compared to those observed in the skull. The morphological differentiation, expressed through PC1 and PC2, underscores the functional and perhaps ecological diversity among the strains. Noteworthy is the distinct mandible shape of the WR strain, separating it from the SD and WA strains. This could point towards different dietary habits, prey–predator dynamics, or other ecological and environmental factors. The significant allometry observed, though lesser than in the skull, still plays a pivotal role in shaping the mandible morphology, underscoring the role of size in functional and ecological adaptations.

Kryštufek et al., in their study on bandicoot rats, noted that contrary to the skull, the mandibular shape did not group species according to taxonomic affiliation [16]. According to the results of the principal component analysis in this study conducted on bandicoot rats, major mandibular features delimiting the two groups from each other are the shapes of the angular, coronoid, and alveolar processes. Taylor et al. [26] reported that there is a more significant distinction between some species in the mandible of African laminate-toothed rats. They also stated that although species differences were significant in their study, approximately 10% of the samples were misclassified as a result of canonical variance analysis. In a recent study, it was observed that the mandible proved successful in distinguishing between rat strains. According to the result of PC1, which explains the highest variation, the most prominent shape variation in the mandible was observed to transition from a slender, elongated structure to an oval one. It was noted that the mandible of WAG/Rij rats exhibited a completely distinct shape from other strains based on the PC1 result.

In recent years, authors have carried out different studies on the shape analysis of the mandible. In his study, Hadžiomerović [27] examined the mandibular shape variations of the red fox and golden jackal and could distinguish these two species in this way. Differentiation between species could be made in different sheep by using geometric morphometrics on the mandible [28,29]. In our study, the mandible was also taxonomically distinctive between the three different rat strains.

In the realm of dental morphology, our findings spotlight unique shape variations among the rat strains. The tooth shape variance, captured by PC1 and PC2, offers insights into the potentially adaptive features among the rat strains. The distinct tooth shape of WR rats, as opposed to the similar shapes in SD and WA rats, could be indicative of dietary specializations or adaptations to specific ecological niches. The role of size, though significant, is less pronounced in influencing tooth shape variations, pointing towards other factors like genetics, diet, or the environment playing substantial roles.

The differences in their head skeleton morphology demonstrated above may affect the suitability of individual rat strains for biomedical experiments. However, it is also worth taking into account the intra-strain variability that occurs among individuals from different vendors [30].

In his study, Maga (2015) utilized the geometric morphometric method to investigate the skull shape and size variation in inbred strains [31]. The study revealed that no single principal component (PC) explained more than 12% of the phenotypic variation. A notable finding was the shape variations of the neurocranium. However, in the current study’s analyses, it was observed that the PC values explained more variation. This disparity could be attributed to the fact that Maga (2015) exclusively used inbred strains in his study. Inbred samples tend to exhibit a greater morphological similarity, which may result in a milder overall shape variation and, consequently, the lower variation explained by the principal components.

In a study by Pallares, the mandibular and cranial shape variation of mice subspecies (*M. m. musculus* and *M. m. domesticus*) was examined, confirming skull and mandible shape differences between these animals [32]. The principal component (PC1) that explained the most variation represented a wider and higher skull shape. In shape analysis studies on mouse mutants and wild types, Lieberman reported that crania with small brains, long faces, and retroflexed cranial bases were distinctive [33]. Unger described in his study using the Longshanks mouse that Longshanks’ skulls became longer, flatter, and narrower in a stepwise process [34]. In a study conducted on three different rat strains, similar shape variations to previous studies were obtained. The most significant change in the principal component (PC1), which explained the highest shape variation, was characterized by the skull being more elongated or oval-shaped. The observed shape variations in the rat skull, particularly the elongation or oval shape change represented by PC1, align with previous findings in the literature. This consistent pattern across studies suggests a morphologically significant aspect of rat skull evolution and variation. The elongation or ova shape change may be indicative of adaptations related to various factors such as diet, locomotion, or an ecological niche. For example, an elongated skull shape could be associated with changes in feeding behavior or dietary preferences, where specialized feeding habits may require modifications to the skull to accommodate different chewing or biting mechanisms. Additionally, the oval shape change could be linked to biomechanical factors related to locomotion. Rats with elongated or oval-shaped skulls may have adaptations that enhance their agility, speed, or maneuverability in their specific environments. These adaptations could include changes in muscle attachment points or skull sutures that allow for more efficient movement. Furthermore, the consistency of this shape change across species, domestic and wild, or between strains suggests that it may be a fundamental aspect of rat skull morphology that is subject to strong evolutionary pressures. Understanding these shape variations and their functional implications can provide valuable insights into the evolutionary history and ecological adaptations of rats and other rodent species.

## 5. Conclusions

This study found that the skull, mandible, and teeth were distinctive for different rat strains in geometric morphometric analyses using two-dimensional images. In particular, shapes of WAG/Rij rats were separated from other rat strains as a result of the principal component analysis. In the canonical variates analysis results, it was seen that all rat strains were separated. Based on these results, it can be said that geometric morphometry is a valuable tool for assessing morphological differences related to population similarity. This analysis technique has a higher degree of accuracy than traditional analytical methods and the results are more statistically inclusive. This method can be used in many shape analysis studies, especially in science branches such as veterinary anatomy and taxonomy.

## Figures and Tables

**Table 1:** Comparison of centroid size for the ventral view of the skull, labial side of the mandible, and occlusal surface of the upper molars for three strains (ANOVA).

Data	Pair	Difference	SE	Q	*p*-Value
Skull	SD-WA	132.49	50.7039	2.613	0.1662
	SD-WR	750.7664	50.7039	14.8069	<0.01
	WA-WR	618.2764	49.8794	12.3954	<0.01
Mandible	SD-WA	137.1119	50.2469	2.7288	0.1422
	SD-WR	733.8179	50.2469	14.6043	<0.01
	WA-WR	596.7059	50.2469	11.8755	<0.01
Teeth	SD-WA	27.3805	21.8871	1.251	0.653
	SD-WR	175.635	22.6048	7.7698	<0.01
	WA-WR	148.2545	21.8871	6.7736	<0.01

SE: Standard error of the difference; Q: Difference/SE.

**Table 2:** Comparison of centroid size for the ventral view of the skull, labial side of the mandible, and occlusal surface of the upper molars for three strains (ANOVA).

Data	Source	DF	Sum of Square	Mean Square	F Statistic	*p*-Value
Cranium	Groups (between groups)	2	5,049,977.158	2,524,988.579	63.4303	<0.01
	Error (within groups)	44	1,751,520.212	39,807.2775		
Mandible	Groups (between groups)	2	4,871,180.276	2,435,590.138	60.2929	<0.01
	Error (within groups)	45	1,817,818.349	40,395.9633		
Teeth	Groups (between groups)	2	253,124.045	126,562.0225	17.6918	<0.01
	Error (within groups)	41	293,302.0438	7153.7084		

**Table 3:** Results of the Canonical Variate Analysis for the different rat strains.

		Procrustes Distance		*p* Value for Procrustes Distance	
		SD	WA	SD	WA
Skull	WA	0.0201		<0.0001	
	WR	0.0445	0.0334	<0.0001	<0.0001
Mandible	WA	0.0366		<0.0001	
	WR	0.0564	0.0684	<0.0001	<0.0001
Teeth	WA	0.0507		0.0035	
	WR	0.0648	0.0753	<0.0001	<0.0001

Wistar Albino, WA; Sprague Dawley, SD; WAG/Rij rats, WR.

**Table 4:** Classification report.

		Classification		Misclassification	
		SD-WA			
Cranium		SD	WA	SD	WA
	SD	15	0	6	9
	WA	0	16	9	7
		SD-WR			
		SD	WR	SD	WR
	SD	15	0	14	1
	WR	0	16	2	14
		WA-WR			
		WA	WR	WA	WR
	WA	16	0	15	1
	WR	0	16	1	15
Mandible		SD-WA			
		SD	WA	SD	WA
	SD	16	0	12	4
	WA	0	16	4	12
		SD-WR			
		SD	WR	SD	WR
	SD	16	0	12	4
	WR	0	16	4	12
		WA-WR			
		WA	WR	WA	WR
	WA	16	0	12	4
	WR	0	16	4	12
Teeth		SD-WA			
		SD	WA	SD	WA
	SD	14	0	6	8
	WA	0	16	8	8
		SD-WR			
		SD	WR	SD	WR
	SD	14	0	13	1
	WR	0	14	0	14
		WA-WR			
		WA	WR	WA	WR
	WA	16	0	14	2
	WR	0	14	1	13

## Data Availability

The data presented in this study are available on request from the corresponding author.

## Appendix A

Ventral aspect of the skull

1. Caudal border of the upper incisor alveolus
2. Lateral border of the upper incisor alveolus
3. Rostralmost point of the palatine fissure
4. Caudalmost point of the palatine fissure
5. Medial extend of the maxilloincisive suture
6. Rostral border of the first molar alveolus
7. Rostralmost point of the second molar (mesial surface)
8. Rostralmost point of the third molar (mesial surface)
9. Caudalmost point of the third molar
10. Infraorbital notch
11. Facial tuber
12. Deepest rostral indentation of the zygomatic arch
13. Deepest caudal indentation of the zygomatic arch
14. Most lateral point of the external acoustic meatus
15. Rostralmost point of the tympanic bulla
16. Caudalmost point of the tympanic bulla
17. Caudalmost point of the occipital condyle
18. Rostralmost point of the foramen magnum
19. Median point of the sphenooccipital suture
20. Median point of the caudal edge of the bony palate

Lateral aspect of the mandible

1. Ventral edge of the lower incisor alveolus
2. Dorsal edge of the lower incisor alveolus
3. Mental foramen
4. Deepest indentation of the interalveolar margin
5. Rostral edge of the first lower molar alveolus
6. Rostral edge of the third lower molar alveolus
7. Apex of the coronoid process
8. Mandibular notch
9. Projection of the mandibular foramen
10. Rostralmost point of the condylar process
11. Caudalmost point of the condylar process
12. Deepest indentation of the caudal edge of the mandibular ramus
13. Angular process
14. Angle of the mandible
15. Facial notch
16. Caudal edge of the mandibular symphysis

Occluseal surface of the upper molars

1–8 tubercles of the M1

9–13 tubercles of the M2

14–18 tubercles of the M3
